# A Bocage Landscape Restricts the Gene Flow of Pest Vole Populations

**DOI:** 10.3390/life12060800

**Published:** 2022-05-27

**Authors:** Aitor Somoano, Cristiane Bastos-Silveira, Jacint Ventura, Marcos Miñarro, Gerald Heckel

**Affiliations:** 1 Servicio Regional de Investigación y Desarrollo Agroalimentario (SERIDA), 33300 Villaviciosa, Asturias, Spain; mminarro@serida.org; 2 Centro de Ecologia, Evolução e Alterações Ambientais (cE3c), Universidade de Lisboa, 1600-214 Lisboa, Portugal; cmsilveira@fc.ul.pt; 3 Departament de Biologia Animal, Biologia Vegetal i Ecologia, Universitat Autònoma de Barcelona, 08193 Bellaterra, Spain; jacint.ventura.queija@uab.ca; 4 Natural Sciences Museum of Granollers, 08402 Barcelona, Spain; 5 Institute of Ecology and Evolution, University of Bern, 3012 Bern, Switzerland; gerald.heckel@iee.unibe.ch

**Keywords:** *Arvicola scherman*, variegated agricultural landscape, cluster structuring, gene flow, graph-based model, isolation-by-distance, landscape genetics, population structure

## Abstract

The population dynamics of most animal species inhabiting agro-ecosystems may be determined by landscape characteristics, with agricultural intensification and the reduction of natural habitats influencing dispersal and hence limiting gene flow. Increasing landscape complexity would thus benefit many endangered species by providing different ecological niches, but it could also lead to undesired effects in species that can act as crop pests and disease reservoirs. We tested the hypothesis that a highly variegated landscape influences patterns of genetic structure in agricultural pest voles. Ten populations of fossorial water vole, *Arvicola scherman*, located in a bocage landscape in Atlantic NW Spain were studied using DNA microsatellite markers and a graph-based model. The results showed a strong isolation-by-distance pattern with a significant genetic correlation at smaller geographic scales, while genetic differentiation at larger geographic scales indicated a hierarchical pattern of up to eight genetic clusters. A metapopulation-type structure was observed, immersed in a landscape with a low proportion of suitable habitats. Matrix scale rather than matrix heterogeneity *per se* may have an important effect upon gene flow, acting as a demographic sink. The identification of sub-populations, considered to be independent management units, allows the establishment of feasible population control efforts in this area. These insights support the use of agro-ecological tools aimed at recreating enclosed field systems when planning integrated managements for controlling patch-dependent species such as grassland voles.

## 1. Introduction

Landscape characteristics are a key factor in the population dynamics of most animal species [1,2,3]. Their relative influence on population connectivity not only depends on patch configuration, but also on behavioural patterns related to the response of individuals to a given landscape [4,5]. Thus, the distribution of each species will be influenced by dispersal capability, resource availability, and the abiotic environment [2,6]. Particularly in those species which depend on patch quality, individuals mainly travel among adjacent habitats, spending as little time as possible in the surrounding matrix [2]. In this way, connectivity between these patches is influenced by the filtering effect of the interstitial matrix, the size of habitat patches and their isolation [7,8]. Specifically, in agricultural areas, landscape is frequently characterised by a patchwork of habitats of varying quality [9]. Depending on the landscape characteristics and the land management of each agro-ecosystem, dispersal and later reproduction can be notably affected [10,11,12]. There have been several population genetics studies on patch-dependent species aiming to better understand how agricultural landscapes determine gene flow [13,14,15]. They have shown that landscape features can both restrict and facilitate the movement of individuals in natural populations. Where the landscape between habitat patches severely restricts dispersal, genetic drift and mutation lead to an overall patchy pattern of genetic variability [13,16]. By contrast, within well-connected habitats, genetic homogeneity can be prevented by species-specific dispersal limitations, leading to a continuous spatial pattern of isolation-by-distance [17,18]. 

Small mammals may provide valuable ecosystem services in agricultural areas: being a key component of the food web and acting as pollinators or seed dispersers [19]. Voles, in particular, can incorporate organic matter into the soil, improving aeration and infiltration and providing suitable sites for seed germination [20]. Studying the population connectivity of rodents in agriculture ecosystems is thus of considerable value. Several genetic studies have highlighted that dispersal is often restricted in rodent species inhabiting agro-ecosystems because of agriculture intensification and the resulting reduction in natural habitats [13,14,15,21]. Maintaining and increasing landscape complexity benefits multiple species at the same time by providing many different ecological niches [22]. However, rodents can also act as crop pests and they may be reservoirs of diseases that can affect human health [23,24,25]. In this scenario, increased landscape heterogeneity would benefit humans and agricultural production through the negative effect it would have on the population dynamics of rodent pests [12,26,27]. 

In Western Europe, a general trend for agricultural intensification since the middle of the last century has resulted in a loss of habitat heterogeneity with serious implications for biodiversity and ecosystem functions in agricultural areas [28,29]. The homogenization of agricultural landscapes has increased the occurrence and the severity of grassland vole species such as common voles, *Microtus arvalis*, and fossorial water voles, *Arvicola scherman* (formerly fossorial form of *A. terrestris* [30], which may spread unimpeded and reach population averages of 500 individuals per hectare, and up to 1000 voles/ha during population peaks [31,32]. Both cyclic vole species are considered serious agricultural pests and a human health hazard in much of their range, and they have consequently received much attention from rodent control organizations [33,34,35]. Specifically, fossorial water voles are considered one of the most serious agricultural pests in grasslands and orchards across France, Switzerland, Germany, and Spain [36]. This subterranean species lives throughout mountainous areas, where landscapes are now mainly characterised by pasture predominance and forest scarcity or absence [37]. Spatial demographic and genetic structural analyses of fossorial water voles have shown that there is often unimpeded dispersal and hence merged and synchronised populations in these homogeneous landscapes [4,18,38]. Consequently, the multiannual fluctuations in the density and the outbreaks of this species may occur at the regional scale [4,32]. 

Fossorial water voles may reach relatively high population densities in crops and grasslands in Atlantic NW Spain, being considered a pest species in this area since the 17th century [39]. However, in contrast to many European mountainous regions, this agricultural area is characterised by a highly variegated landscape where field boundaries such as hedgerows and woodlands are frequent between agricultural plots. This traditional configuration is based on a fine-grained mosaic of different land-use plots, and it is the predominant farming management approach in this area [40]. This enclosed field system, or bocage landscape, favours biodiversity [41], including both terrestrial and avian predators of voles, and it markedly differs from modern agricultural landscapes in other parts of Western Europe [42]. Concerning pest management, a prime asset of hedgerows and woodlots are that they hamper, at least partially, fossorial water vole dispersal across the landscape [12]. In fact, among specific and environmentally benign control practices [43], limiting the population spread of fossorial water voles by increasing landscape heterogeneity has recently been highlighted as a promising agro-ecological tool [12]. However, to our knowledge, there is no empirical study that has tested the influence of a highly-complex agricultural landscape on the distribution of genetic variation and the gene flow of pest voles. 

Using a landscape genetic approach based on the spatial distribution of neutral genetic variation at both the population and the individual level, we tested the hypothesis that a bocage landscape influences patterns of genetic structure in fossorial water voles at the local scale. Higher levels of genetic differentiation may therefore be expected in this variegated landscape compared to those that are open and more functionally connected for vole pests [18,44]. Genetic data from ten populations (demes) were thus used, together with high-resolution land-cover and land-use information, to (1) examine population structure and quantify genetic variation in these fossorial water voles, and (2) uncover the influence of the landscape on genetic structure. These insights can be useful to better understand the impact of different agro-ecosystems on the population spread of grassland voles [17,45]. Likewise, knowing how the landscape affects successful dispersal of pest species may enhance management policies aimed at ensuring food security and the prevention of the spread of disease [46,47]. 

## 2. Material and Methods

### 2.1. Study Area

**Figure 1:**
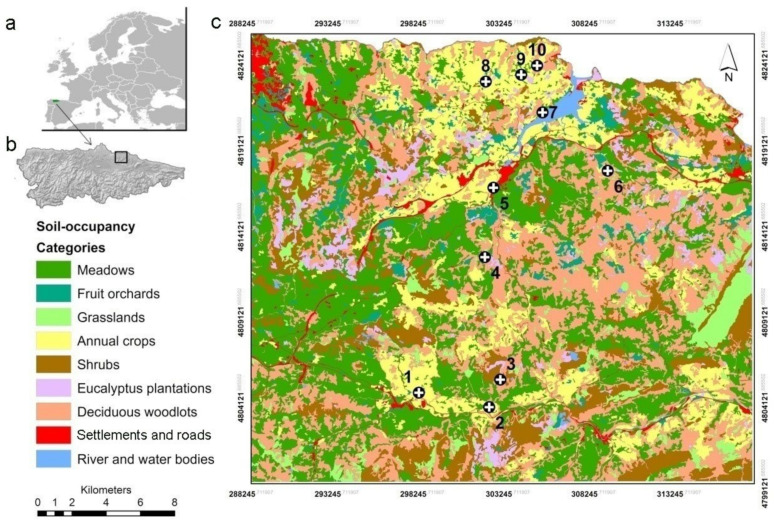
Location of Asturias (NW Spain) (**a**) and the study area in this region, framed by a black square (**b**). Map of soil occupancy (Land Cover and Use Information System of Spain, SIOSE) (**c**). The sampled orchards are indicated by numbers: 1, Vegadali; 2, Ceceda; 3, Fresnadiello; 4, Poreño; 5, Serida; 6, Priesca; 7, Rozada; 8, Oles; 9, Teleña; 10, Marina.

The study was conducted in an agricultural area covering approximately 120 km^2^ around the municipalities of Villaviciosa (43°38′ N, 5°26′ W) and Nava (43°21′ N, 5°30′ W) (Asturias, NW, Spain) (Figure 1a,b). The agro-ecosystem is characterised by a highly variegated landscape, containing a fine-grained mosaic of orchards, hay meadows, livestock pastures, annual crops (mostly silage maize), eucalyptus plantations, settlements, and semi-natural woody vegetation patches, mostly temperate broad-leaved forest, heathland patches, and riverine forest. Crop activity is developed in small plots separated by minimally-managed hedgerows or small woodland patches. The most favourable habitats for fossorial water voles in this area (meadows, grasslands and fruit orchards [48] show evergreen and dense grass coverage all year around because of relatively high rainfall and fertile soils. The relief of the area is generally gentle with moderate hills up to 500 m a.s.l. An estuary and a four-lane highway (A-8) divide some sampling points in the study area. Cider apples are the most important fruit crop in this area, and fossorial water voles are considered a key pest species in orchards, and their demographic densities need to be controlled in accordance with article 15 of the Law 43/2002 of plant health [49]. There is no specific information on vole densities in the study area, but the relatively high *A. scherman* abundance we observed in apple orchards and the associated severe crop damage are compatible with a population outbreak. 

### 2.2. Specimen Collection

Fossorial water voles were collected throughout 2011–2012 in 10 semi-intensive apple orchards covering areas from 1 to 7.6 ha and at altitudes from 3 to 270 m a.s.l. A single sampling was conducted in each sampled plot. Snap traps (Topcat^®^ Andermatt Biocontrol, Grossdietwil, Switzerland) were placed in galleries for five consecutive days to collect specimens. A variable number of traps was set in each sampling plot according to surface signs of activity, using one trap per group of earth mounds observed. Shortly after capture, each specimen was cryopreserved at −20 °C until necropsy. Then, a sample of muscle tissue was preserved in 70% ethanol at −20 °C. Specimens gathered from each orchard were georeferenced and initially considered as belonging to independent populations (demes). Further favourable inhabited patches were found to exist close to our sampled demes. The recommendations of the Directive of the European Parliament and the Council on the Protection of Animals Used for Scientific Purposes [50] (Directive 2010/63/UE 2010) were considered in all procedures.

### 2.3. Graph-Based Landscape Model 

The assessment of landscape configuration through a graph-based model can provide a spatial representation of a habitat which offers valuable information for the study of connectivity according to the biology and the ecology of fossorial water voles [7,51]. Habitat patches were represented by polygons of soil occupancy with a 5 m^2^ resolution, extracted from the Land Cover and Use Information System of Spain (SIOSE [52]) and analysed in a vector-based geographic information system (GIS). Soil-occupancy polygons were classified into nine categories: meadows, fruit orchards, pastures or grasslands, annual crops, shrubs, eucalyptus plantations, deciduous woodlots, settlements and roads, and water bodies (Figure 1). A cost-distance model (resistance layer) was developed according to the suitability of the patches as habitats and their permeability for fossorial water voles [53,54]. A multivariate resistance surface was developed in which each cell of the layer (5 m^2^) was attributed a cost value (CV) depending on each soil-occupancy category. Since there is no information on densities in this area that would have allowed a study of the influence of the landscape on population spread processes, these CVs were based on cost scenarios which had been compared to demographic indicators derived from field records in agricultural landscapes from France [12,55], complemented by data on the species’ habitat preference in NW Spain [48]. Cost values were assigned to each soil-occupancy category, in which a greater inter-patch cost distance would indicate increased demographic asynchrony because of less permeability [12]: CV = 1: hay meadows, grasslands and fruit orchard; CV = 25: annual crops and shrubs; CV = 50: settlements and roads; CV = 1000: deciduous woodlots, eucalyptus plantations and water bodies. 

To evaluate connectivity between pairs of individuals and demes, we used the cost-distance model to quantify (in metres) “effective distances”. Least-cost paths (LCPs), defined as modified Euclidean distances based on resistance surface (ESRI 1996) were calculated to establish ecologically relevant links [53]. For any given movement from cell N_i_ to cell N_i+1_, the cumulative cost is calculated as the cost to reach a cell N_i_ plus the average cost to move through cell N_i_ and cell Ni_+1_ in which the algorithm considers eight neighbour cells and allows diagonal movements [56]. To estimate “resistance distances” we created a model $(\;\sum_{i=1}^{n}\left[m_{i}CV_{i}\right]$) which considered the cost value of each category (*CV_i_)* and the total length (in metres) of the crossed patches (*m_i_*). “Resistance distance” was calculated both for pairs of individuals and demes. A digital elevation model (based on a 5 m scaled grid) was also implemented to consider a potential increase of distance because of orography. Additionally, a straight line or a Euclidean distance was calculated between individuals and pairwise demes. The centre of the plot was considered as the location reference in between-demes distances.

Information on landscape-level processes in *A. scherman* should be considered to assess link probabilities between demes [6,54]. In that sense, and according to the Ratio of Optimal to Marginal Patch Area (ROMPA) hypothesis, the higher the ratio of suitable habitats (meadows, orchards and grasslands) to the total land area (SH/TL), the higher the link probabilities between demes [32,57]. Thus, the SH/TL ratio was calculated for the area of intersection between two circular buffers around each orchard that considered the centre of the plot as the origin and the Euclidean distance between them as the radius. We define “landscape suitability” as the SH/TL ratio between sampling points corrected by Euclidean distance ([SH/TL]/Euclidean distance). Spatial analysis and GIS data management were conducted with ArcGis 10.3 [56].

### 2.4. Microsatellite Genotyping

Genomic DNA was extracted using a GeneMatrix Tissue Purification Kit (EURx). PCR-amplification of 12 microsatellite loci was carried out in two multiplex panels: panel 1: AV3, AV8, AV11, AV15, AT2 and AT24; and panel 2: AV12, AV14, AV13P, AT9, AT13, and AT22 [38,58,59]. An AV13 reverse microsatellite primer was labelled with a “pig-tail” to increase the accuracy of genotyping. Forward primers were labelled using FAM, NED, PET and VIC fluorochromes. Genotyping was conducted in 10 µL reactions. Each reaction contained 1 µL primer mix, 5 µL GoTaq Green Master Mix (containing *Taq*-polymerase, MgCl_2_, dNTPs and PCR buffer) and 1.5 µL DNA (50 ng/µL). The PCR profile had an initial denaturation step at 93 °C for 2 min, then thirty cycles of 30 s at 91 °C, 30 s at 57 °C and 30 s at 74 °C. The PCR ended with an elongation phase at 74 °C for 10 min and then the PCR products were stored at 4 °C. Fragment separation was carried out on an ABI 3130 sequencer and the genotypes were scored using Genemapper software 3.7 (Applied Biosystems) against the internal LIZ 500 size standard.

### 2.5. Genetic Diversity, Structure, and Differentiation

The software Convert 1.3 [60] was used to prepare the input files for microsatellite data analyses. Genotypic linkage disequilibrium (LD) and deviations from Hardy–Weinberg Equilibrium (HWE) were tested by locus and by deme using exact tests implemented in the software Genepop 4.2.2 [61]. Null alleles were checked for using Micro-Checker [62]. Expected- (H_E_) and observed (H_O_) heterozygosity levels [63] were calculated with the program Genetix 4.05 [64]. The number of alleles (N_A_), allelic richness corrected for minimum sample size (A_R_), and inbreeding coefficients (F_IS_) for each population, along with fixation index (F_ST_) for each pair of demes and overall were calculated using the software Fstat 2.9 [65]. Bootstrap resampling was conducted in this software to calculate mean and 95% confidence intervals for F_ST_.

To evaluate potential recent migration between populations we used the software Geneclass 2.0 [66], with the settings recommended for when not all source populations are sampled (direct likelihood L_home). The probability of an individual being a resident was assessed by 10,000 Monte Carlo simulations using the [67] algorithm. The probability of type I error was set to *p* < 0.01 and the default frequency of missing alleles to 0.01.

Genetic subdivision within the area studied was assessed using non-spatial and spatial clustering methods. First, we used the non-spatial model-based Bayesian clustering method implemented in Structure 2.3.4 [68]. This software considers multilocus genotypes, and it attempts to minimise HW disequilibrium by assigning them to a number of genetic clusters (*K*). Structure was run with ten repetitions of 500,000 Markov Chain Monte Carlo (MCMC) iterations following a burn-in period of 100,000 steps, the admixture model, correlated allele frequencies, and sampling locations as prior information. The range of *K* was set to include the total number of sampled demes (*K* = 10), and analyses for each *K* were run 10 times. Structure Harvester [69] was used to collate the results and infer the best-supported *K* using both posterior probability of the data for a given value of *K* [Ln P (*K*)] and its rate of change (∆*K*). Given that the geographical position of samples may have an impact on the outcome of clustering approaches [70,71], Geneland 4.0.5 [70] was used to infer the genetic structure in the study area, also taking into account the sampling coordinates of the individuals. Ten MCMC runs were conducted, each comprising 500,000 iterations with a thinning of 1000 and a maximum rate of Poisson process fixed to 100. The number of clusters, *K*, was allowed to vary between 1 and 10. MCMC convergence was assessed by comparing the number of clusters across replicate runs, with a single run chosen for presentation on the basis of the mean posterior probability as a criterion. 

The distribution of genetic variation and the significance of differentiation was also examined through the analysis of molecular variance (AMOVA) based on the clusters inferred by Structure and Geneland [71] (Meirmans 2012). A three-level AMOVA (individual, deme, and inferred cluster) was conducted in Arlequin 3.5 with 10,000 permutations using an F_ST_-like estimator under the infinite alleles model (IAM) [72,73]. We tested how much genetic variation was explained by differences between individuals within demes, between demes within a cluster, and between clusters, and we tested the significance with 10,000 permutations. 

### 2.6. Effect of the Landscape on Genetic Structure

Mantel tests [74], with 1000 randomizations for independence of matrices of pairwise dissimilarities, were conducted to test genetic differentiation between demes influenced by landscape variables (isolation by distance, IBD; isolation by resistance, IBR; or landscape suitability) using the software Isolation by Distance [75]. For this, least major axis regressions [76] were used considering pairwise decimal logarithms of landscape variables and genetic distances between pairs of demes after linearisation of F_ST_/(1 − F_ST_). In addition, partial Mantel tests were used in a causal modelling framework to identify which variable best explained the observed genetic structure [71,77]. Thus, the correlation of genetic differentiation (F_ST_) for each pair of demes and a matrix describing hierarchical structure was examined using a third matrix of each landscape variable as an indicator [72,78]. Likewise, partial Mantel tests were conducted for each landscape variable partialling out the effect of Euclidean distance, resistance distance, landscape-suitability values and cluster membership. This matrix of cluster membership was generated using a distance value of 1 for same-cluster demes and of 0 for different-cluster demes.

Genetic spatial autocorrelation analysis at the individual level was conducted in Genealex 6.5 [79]. The genetic relatedness coefficient (*r*) was calculated comparing the genetic similarity of pairs of multilocus genotypes from individuals within each distance class (also known as interval) with the mean genetic similarity of all pairs of individuals [80]. In total, 999 random permutations of genotypes were used to generate the null distribution for genetic distance, and 1000 bootstrapping replicates of pairwise comparisons for each distance class were used to generate 95% confidence intervals for *r* [80,81]. Distance classes are bounded by a lower and an upper distance (e.g., 0–100 m) with all pairs of individuals separated by distances that fall within these bounds included in the calculation of *r* for that distance class [81]. A positive relatedness coefficient would represent higher than expected genetic similarity within a given distance-class relative to the entire population. In contrast, no difference from zero or negative coefficients would represent less genetic similarity than the mean relatedness in the sample [80,81].

## 3. Results

### 3.1. Genetic Diversity

A total of 137 fossorial water voles (71 males, 66 females) were successfully genotyped at 12 microsatellite loci (Table S1). The number of alleles per locus ranged from 3 to 18 with a mean observed heterozygosity per locus of 0.602 and a mean expected heterozygosity of 0.732 (details Table S1). The loci AV13P and AV14 showed significant linkage disequilibrium across all demes (*p* < 0.001). As a result, locus AV14 was excluded prior to further analyses being performed as it had a lower number of alleles than AV13P. 

The number of genotyped voles per deme ranged between 11 and 18 (Table 1). Allelic richness ranged from 3.40 to 5.40 (Table 1), with an average value of 4.26. All but two demes (Serida and Oles) conformed to HWE (Table 1). These two demes showed significant heterozygosity deficiency associated with very high inbreeding coefficients (Serida: F_IS_ = 0.183, *p* < 0.001; Oles: F_IS_ = 0.180, *p* < 0.001). The software Geneclass indicated seven first-generation immigrants in the studied demes: Vegadali (1), Ceceda (1), Serida (1), Priesca (2), Rozada (1) and Oles (1). The immigrant in Ceceda was a home-assigned immigrant, meaning that it was genetically closer to its deme of capture than others sampled. Priesca and Rozada received immigrants from demes genetically closer to Teleña and Marina. 

### 3.2. Population Structure in A. scherman

**Figure 2:**
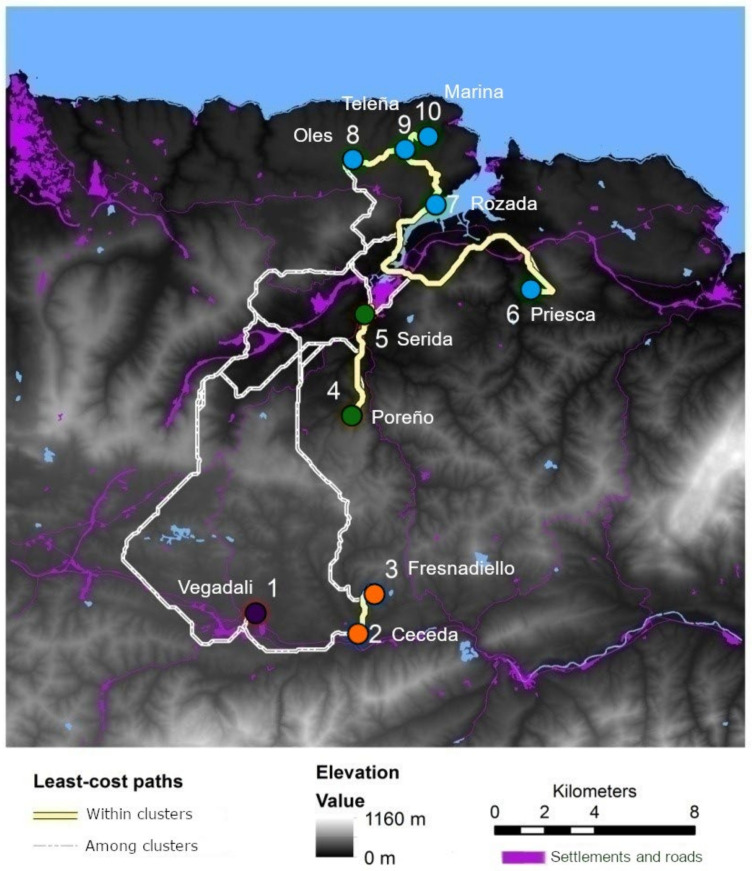
Fossorial water vole demes in Asturias (NW Spain) with the same colour indicating the estimated population kinship based on the mode of posterior probabilities inferred by Geneland and according to individual kinship coefficients inferred by Structure for *K* = 4. The sampled demes are indicated by name and number. Least-cost paths are represented within and between inferred genetic clusters (yellow and white lines respectively). The main relief features, settlements and roads are also shown.

**Figure 3:**
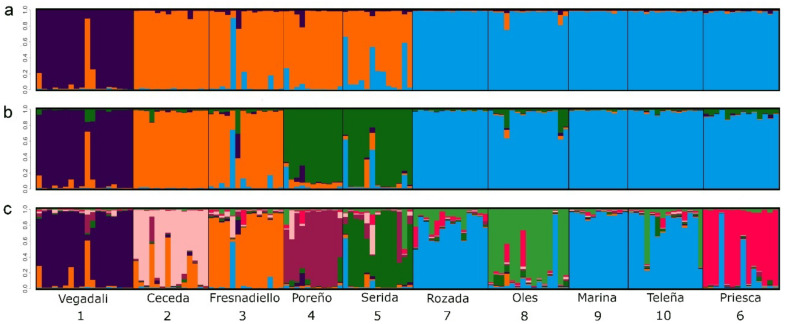
Individual kinship coefficients inferred from Bayesian inference of genetic clustering using Structure analysis across ten demes of fossorial water voles from Asturias (NW Spain) for the number of clusters *K* = 3 (**a**), *K* = 4 (**b**) and *K* = 8 (**c**). Each single vertical line represents an individual. A standard admixture model was used that included sampling locations as prior information.

The pairwise tests of genetic differentiation were significant (*p* < 0.05) for 38 of 45 pairs of fossorial water vole demes. Estimates of F_ST_ ranged between 0.003 and 0.281 (Table S2). The estimate of F_ST_ across all sites and loci was 0.168 (bootstrapping interval: 0.128–0.219). The estimation of the number of genetic clusters with Geneland across all runs yielded *K* = 4 (Figure S1a) with a largely north-south distribution of clusters (Figure 2). The non-spatial approach in the structure suggested likelihood peaks for *K* = 3 (Figure 3a) and *K* = 8 (Figure 3c), with a steady increase in Ln P (*K*) from *K* = 1 to *K* = 8 (Figure S1b,c). Clustering outcomes of individuals at *K* = 4 (Figure 3b) matched those of Geneland, and increasing *K* from 3 to 8 showed highly consistent clustering of individuals with consecutive subdivision of larger clusters. Hierarchical AMOVAs demonstrated that increasing the number of clusters from 3 to 8 (i.e., keeping more and more demes separate) resulted in an increase in the proportion of genetic variation explained (Table 2). At the same time, the proportion of genetic variation explained by different demes in clusters dropped from 8.9 to 2.8%, although it remained significant even for *K* = 8 (Table 2). This suggested that genetic subdivision between demes in the bocage landscape is extensive and spatial proximity may explain some of the differentiation in this system. 

### 3.3. Effect of the Bocage Landscape on Genetic Structure

**Figure 4:**
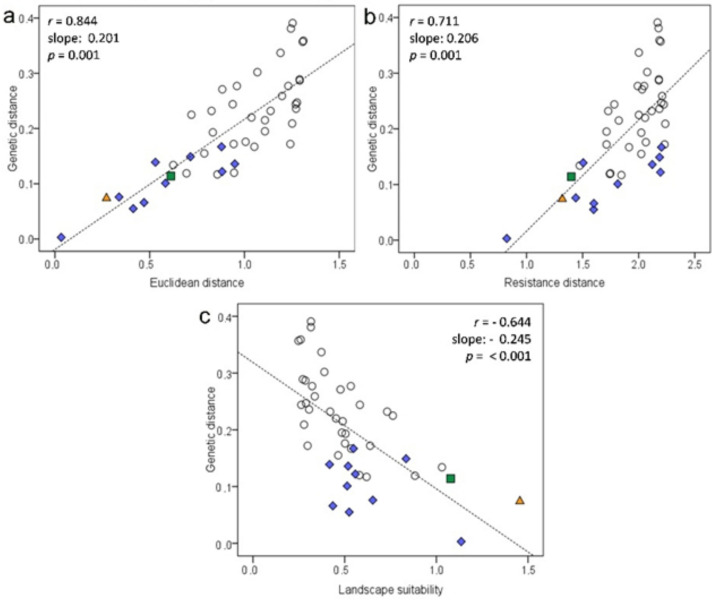
Isolation-by-distance (IBD) (**a**), Isolation-by-resistance (IBR) (**b**) and relationship between landscape suitability and genetic distance (**c**) in fossorial water vole demes in Asturias (NW Spain). The graphs show genetic distance (F_ST_/1 – F_ST_) versus logarithmic Euclidean distance, logarithmic resistance distance and logarithmic landscape suitability ((SH/TL)/Euclidean distance) for all possible pairwise comparisons between the ten demes. Comparisons within the same genetic cluster according to Geneland are shown in colour: *orange triangle*, Fresnadiello and Ceceda; *green square*, Poreño and Serida; *blue diamond*, Priesca, Rozada, Oles, Teleña, and Marina.

Euclidean and effective distances between demes ranged from 1.08 to 20.48 km (Mean 10.54 ± 6.03 SD) and from 1.50 to 42.23 km (Mean 20.28 ± 12.51 SD), respectively (Table S2a). Resistance distances between demes varied from 6.67 to 172.63 (Mean 100.84 ± 50.49 SD) (Table S2b). A relatively low ratio (%) of suitable habitat was overall observed (31.58 ± 10.04 SD), even within inferred clusters (24.40, 48.98 and 53.41%, respectively, in clusters from north to south) (Table S2b). A Mantel test for Euclidean distance between fossorial water vole demes and genetic differentiation showed a significant positive relationship and thus a strong pattern of IBD (Figure 4a). Incorporating habitat information into spatial distances (IBR) also revealed a strongly significant positive relationship (Figure 4b), albeit those genetic distances were no better explained by resistance than by simple Euclidean distances. Furthermore, landscape suitability data was strongly inversely related to genetic differentiation between demes (Figure 4c). Partial Mantel tests (Table 3) demonstrated that the IBD relationship was robust enough to discount the effect of IBR or landscape suitability, while the latter two factors were not significant without IBD (Table 3). Considering eight potential genetic clusters in partial Mantel tests did not lead to stronger IBD relationships than no clustering (Table 3). 

**Figure 5:**
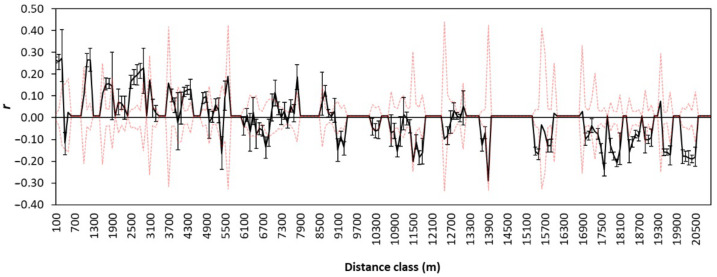
Spatial autocorrelation (*r*) for Euclidean distance class sizes of 100 m in fossorial water voles from Asturias (NW Spain). 95% confidence intervals for estimates of *r* from 1000 bootstrap replicates on pairs of individuals are shown with error bars; 95% confidence intervals based on 999 random permutations for the null hypothesis of no spatial autocorrelation are shown with dashed red lines.

Fossorial water voles within sampling locations were significantly more closely related to each other than random individuals (Figure 5). Spatial autocorrelation analyses also showed increased relatedness at distance classes 1000–1300 m and 2500–2800 m. Larger distance classes showed only negative deviations from random relatedness between individuals, which is consistent with the strong population structure between demes (Figure 5). Overall, there was no evidence of spatial autocorrelation between individual voles in this bocage landscape (*p* = 0.736). 

## 4. Discussion

We present here the first landscape genetics study for an agricultural pest rodent in a highly variegated landscape, testing the hypothesis that landscape affects successful dispersal and thus influences the genetic structure pattern at the local scale. The results showed a strong IBD pattern supported by a high and a significant genetic correlation between fossorial water voles at smaller geographic scales. This distance-dependence of dispersal on genetic differentiation can also be expected at larger geographical scales, where the direct exchange of migrants is unlikely to occur [17,38]. However, autocorrelation patterns showed negative deviations from random relatedness between individuals at distances over 2800 m. No evidence of an IBD pattern at the larger scale was observed, unlike what has been reported in other agricultural landscapes for common voles [17,21] or specifically for fossorial water voles [38]. Rather, high genetic differentiation indicated a hierarchical pattern of up to eight potentially relevant clusters. This distribution of genetic variation would indicate a metapopulation-type dynamic in these fossorial water voles [82,83]. These clusters can thus be considered largely independent demographic units whose size depends mainly on local birth and death rates [84,85]. Stochastic processes of patch occupancy are also likely to take place in this area [86], leading to a chaotic genetic patchiness and contributing to determining this genetic structure [47]. Complex patterns of genetic heterogeneity have been observed previously in rodents [47,58,87]; although, to our knowledge, this is the first time this kind of genetic structure has been reported for a pest vole species inhabiting an agro-ecosystem. 

This metapopulation structure may be determined by kin clustering such as occurs in northern water voles (semiaquatic form of *A. amphibius*, [58,88]. However, the overall findings of no clear deviations from Hardy–Weinberg equilibrium and low inbreeding coefficients are consistent with the essential nature of dispersal and colonisation of new habitats in fossorial water voles, responding to inbreeding avoidance, mate searching and niche availability [12,18]. Patterns of genetic differentiation in arvicoline rodents may also be affected by resource availability [38,89]. Nonetheless, a loss of genetic variability in these fossorial water voles due to increased variance in reproductive fitness [90] seems unlikely. Mild temperatures, even in winter, and ample food all the year around allow populations to continuously breed and to show a high reproductive potential in this geographic area [36,91]. Because a population outbreak occurred during fieldwork in the whole sampling area, genetic differentiation caused by low dispersal rates during a demographic crash could be also rejected [38]. Therefore, it seems that landscape characteristics and land management of this agro-ecosystem are responsible for determining the dispersal and later reproduction of fossorial water voles [10,11,12].

Landscape configuration is considered a key factor in determining gene flow between fossorial water vole populations [12,37]. Indeed, the strong pattern of IBR and the significant inverse relationship between landscape suitability and genetic differentiation in these fossorial water voles suggests that gene flow was determined to a great extent by the connectivity and the abundance of suitable habitats. Nevertheless, including this habitat information did not explain genetic differentiation better than Euclidean distances at a fine geographic scale. It should be considered that the parameterization of resistance values, based partially on other geographic areas, may not guarantee meaningful information for assessing *A. scherman* movements across this landscape. In this way, it could be advisable to use these genetic data to unbiasedly develop resistance surfaces [92]. The studied bocage landscape showed a relatively low ratio of suitable habitats at the local scale, leading to a large extent of matrix. Thus, the extent of the matrix in which habitats are immersed rather than matrix heterogeneity *per se* seems to have an important effect on determining gene flow in this patch-dependent species [93]. Consecutive dispersal and reproduction during population outbreaks may allow fossorial water voles to spread over 7 km per year in open and functionally connected landscapes [4]. Conversely, a considerable decrease in the number of emigrants as distance increases is likely to occur within variegated landscapes because of the short-distance dispersal in this species (a few hundred metres) [94]. Surrounding ecotonal boundaries may cause individuals to tend to cluster inside the habitat [2,32], although all but two demes showed no significant heterozygosity deficiency, which is associated with inbreeding coefficients. Despite no study on predator pressure having been carried out to date, biological control conducted by both specialised vole predators (*Mustela erminea*, *Mustela nivalis*) and those that are not specialised (*Vulpes vulpes*, *Tyto alba*, *Buteo buteo*) [41,95] may play a notable role in this agro-ecosystem with plentiful resources [2]. This matrix could therefore be considered as a demographic sink, where gene flow of water voles is likely to decline because of short-distance dispersal and increased mortality [96]. 

In homogeneous agricultural landscapes from Western Europe, multiannual fluctuations of density and outbreaks of fossorial water voles occur at the regional scale [4,32] because of unimpeded dispersal [4,18,38]. In contrast, genetic structure in this study indicated that those multiannual fluctuations in density may take place at a sub-population scale in this agro-ecosystem. It was hypothesised that fossorial water voles would show long phases of low-density along with slight fluctuations and sometimes high-density populations in a variegated landscape such as this [32,57]. Further demographic studies over time are strongly recommended to corroborate this hypothesis and to predict population outbreaks. Nonetheless, this study may have direct implications for establishing a management program aimed at increasing crop protection and reducing human health risks [46,47]. Colonisation processes may not be related directly with livestock breeding activity but with a general trend in agricultural abandonment in this area [97]. Scarcely attended meadows and crops would represent highly favourable habitats for fossorial water voles [98]. The identification of sub-populations, which can be considered as independent management units, allows the establishment of reasonable efforts to conduct population control in this variegated landscape [84,85]. According to our results, an overall ratio of landscape suitability <35% could be a constraint on the gene flow of fossorial water voles at distances greater than 2800 m. Ultimately, in the broader sense, these genetic insights empirically support the use of agro-ecological tools which aim to recreate enclosed field systems in order to establish integrated management approaches to control species which depend on habitat features, such as grassland vole populations [12].

## Figures and Tables

**Table 1:** With respect to each deme sampled, the following data are shown: sampling date, geographic coordinates of the apple orchard, plot area (ha), (N) number of fossorial water voles genotyped, number of alleles (N_A_), allelic richness corrected for minimum sample size (A_R_), observed heterozygosity (H_O_), expected heterozygosity (H_E_), inbreeding coefficient (significant cases in bold) and Hardy–Weinberg equilibrium exact test (HWE *p*-value).

Orchard/ Deme	Date		UTMx	UTMy	Area (ha)	N	N_A_	A_R_	H_O_	H_E_	F_IS_	HWE *p*-Value
1-Vegadali	Sep	2011	298049	4804471	2.31	18	6.09	5.35	0.636	0.695	0.087	0.031
2-Ceceda	Dec	2012	302152	4803618	5.95	14	4.82	4.53	0.604	0.629	0.042	0.434
3-Fresnadiello	Jun	2011	302809	4805229	2.84	14	6.00	5.40	0.656	0.692	0.052	0.207
4-Poreño	Jul	2012	301923	4812449	7.58	11	4.64	4.64	0.612	0.614	0.005	0.610
5-Serida	Jun	2011	302393	4816492	7.26	12	4.64	4.47	0.458	0.556	**0.183**	<0.001
6-Priesca	Apr	2011	309079	4817418	2.03	14	3.55	3.44	0.591	0.554	−0.071	0.582
7-Rozada	Mar	2012	305277	4820835	2.99	14	3.91	3.75	0.524	0.542	0.034	0.467
8-Oles	Jun	2011	301922	4822568	4.55	15	4.00	3.40	0.491	0.595	**0.180**	<0.001
9-Teleña	Jul	2012	304030	4823030	1.13	14	3.82	3.75	0.656	0.612	−0.075	0.545
10-Marina	Jul	2012	304955	4823559	1.00	11	3.91	3.91	0.595	0.587	−0.015	0.528

**Table 2:** Three-level hierarchical AMOVA according to hierarchical structuring results (*K* level) for fossorial water voles in Asturias (NW Spain).

			Percentage of Variation	Fixation Indices	*p-*Value
Between individuals within demes	3	127	3.65	0.046	0.011
	4	127	3.68	0.046	0.011
	8	127	3.78	0.046	0.008
Between demes within clusters	3	7	8.95	0.100	<0.001
	4	6	7.52	0.085	<0.001
	8	2	2.84	0.033	<0.001
Between clusters	3	2	10.91	0.109	<0.001
	4	3	11.81	0.118	<0.001
	8	7	14.71	0.147	0.009

**Table 3:** Partial Mantel tests for IBD, IBR, landscape suitability and 8-cluster structuring in fossorial water vole demes in Asturias (NW Spain). For each case, the regression coefficient (*r*) and probability (*p*) for tests of significance based on 1000 permutations are shown.

Test		Corrected by	*r*	*p-*Value
IBD	Euclidean distance	Resistance distance	0.630	0.001
		(SH/TL)/Euclidean distance	0.733	0.002
		8-cluster structuring	0.770	0.001
IBR	Resistance distance	Euclidean distance	0.093	0.234
		(SH/TL)/Euclidean distance	0.520	0.013
		8-cluster structuring	0.568	0.004
Landscape suitability	(SH/TL)/Euclidean distance	Euclidean distance	−0.151	0.175
		Resistance distance	−0.246	0.052
		8-cluster structuring	−0.513	1.000
Clusters	8-cluster structuring	Euclidean distance	0.401	0.016
		Resistance distance	0.386	0.015
		(SH/TL)/Euclidean distance	0.523	0.001

## Data Availability

Graph-based landscape information and microsatellite genotyping data are available via the Dryad Digital Repository https://doi.org/10.5061/dryad.h9w0vt4k5 (accessed on 6 May 2022) [99].

## Supplementary Materials

The following supporting information can be downloaded at: https://www.mdpi.com/article/10.3390/life12060800/s1, Figure S1: Results from GENELAND analysis; Table S1: Descriptive statistics of microsatellite loci; Table S2: Pairwise F_ST_ − Euclidean/resistance values, and pairwise F_ST_ − landscape suitability/ROMPA (SH/TL) values.

## References

[1] Gilarranz L.J., Bascompte J. (2012). Spatial network structure and metapopulation persistence. J. Theor. Biol..

[2] Driscoll D.A., Banks S.C., Barton P.S., Lindenmayer D.B., Smith A.L. (2013). Conceptual domain of the matrix in fragmented landscapes. Trends Ecol. Evol..

[3] Chiu M.C., Nukazawa K., Carvajal T., Resh V.H., Li B., Watanabe K. (2020). Simulation modeling reveals the evolutionary role of landscape shape and species dispersal on genetic variation within a metapopulation. Ecography.

[4] Berthier K., Piry S., Cosson J.F., Giraudoux P., Foltête J.C., Defaut R., Truchete D., Lambin X. (2014). Dispersal, landscape and travelling waves in cyclic vole populations. Ecol. Lett..

[5] Craig V.J., Klenner W., Feller M.C., Sullivan T.P. (2015). Population dynamics of meadow voles (*Microtus pennsylvanicus*) and long-tailed voles (*M*. *longicaudus*) and their relationship to downed wood in early successional forest habitats. Mammal Res..

[6] Wiegand T., Revilla E., Moloney K.A. (2005). Effects of habitat loss and fragmentation on population dynamics. Conserv. Biol..

[7] Galpern P., Manseau M., Fall A. (2011). Patch-based graphs of landscape connectivity: A guide to construction, analysis and application for conservation. Biol. Conserv..

[8] Gauffre B., Mallez S., Chapuis M.P., Leblois R., Litrico I., Delaunay S., Badenhausser I. (2015). Spatial heterogeneity in landscape structure influences dispersal and genetic structure: Empirical evidence from a grasshopper in an agricultural landscape. Mol. Ecol..

[9] Janova E., Heroldova M. (2016). Response of small mammals to variable agricultural landscapes in Central Europe. Mamm. Biol.-Z. Für Säugetierkunde.

[10] Jacob J., Hempel N. (2003). Effects of farming practices on spatial behaviour of common voles. J. Ethol..

[11] Marchi C., Andersen L.W., Damgaard C., Olsen K., Jensen T.S., Loeschcke V. (2013). Gene flow and population structure of a common agricultural wild species (*Microtus agrestis*) under different land management regimes. Heredity.

[12] Foltête J.C., Couval G., Fontanier M., Vuidel G., Giraudoux P. (2016). A graph-based approach to defend agro-ecological systems against water vole outbreaks. Ecol. Indic..

[13] Howell P.E., Delgado M.L., Scribner K.T. (2017). Landscape genetic analysis of co-distributed white-footed mice (*Peromyscus leucopus*) and prairie deer mice (*Peromyscus maniculatus bairdii*) in an agroecosystem. J. Mammal..

[14] Vera N.S., Chiappero M.B., Priotto J.W., Sommaro L.V., Steinmann A.R., Gardenal C.N. (2019). Genetic structure of populations of the Pampean grassland mouse, *Akodon azarae*, in an agroecosystem under intensive management. Mamm. Biol..

[15] Otero-Jiménez B., Li K., Tucker P.K. (2020). Landscape drivers of connectivity for a forest rodent in a coffee agroecosystem. Landsc. Ecol..

[16] Hamilton G.S., Mather P.B., Wilson J.C. (2006). Habitat heterogeneity influences connectivity in a spatially structured pest population. J. Appl. Ecol..

[17] Gauffre B., Estoup A., Bretagnolle V., Cosson J.F. (2008). Spatial genetic structure of a small rodent in a heterogeneous landscape. Mol. Ecol..

[18] Berthier K., Charbonnel N., Galan M., Chaval Y., Cosson J.F. (2006). Migration and recovery of the genetic diversity during the increasing density phase in cyclic vole populations. Mol. Ecol..

[19] Jones K.E., Safi K. (2011). Ecology and evolution of mammalian biodiversity. Philos. Trans. R. Soc. Lond. B.

[20] Martin B.G. (2003). The role of small ground-foraging mammals in topsoil health and biodiversity: Implications to management and restoration. Ecol. Manag. Restor..

[21] Schweizer M., Excoffier L., Heckel G. (2007). Fine-scale genetic structure and dispersal in the common vole (*Microtus arvalis*). Mol. Ecol..

[22] Herrera J.M., Salgueiro P.A., Medinas D., Costa P., Encarnação C., Mira A. (2016). Generalities of vertebrate responses to landscape composition and configuration gradients in a highly heterogeneous Mediterranean region. J. Biogeogr..

[23] Weber de Melo V., Sheikh Ali H., Freise J., Kühnert D., Essbauer S., Mertens M., Wanka K.M., Drewes S., Ulrich R.G., Heckel G. (2015). Spatiotemporal dynamics of Puumala hantavirus associated with its rodent host, *Myodes glareolus*. Evol. Appl..

[24] Drewes S., Sheikh Ali H., Saxenhofer M., Rosenfeld U.M., Binder F., Cuypers F., Schlegel M., Röhrs S., Heckel G., Ulrich R.G. (2017). Host-associated absence of human Puumala virus infections in northern and eastern Germany. Emerg. Infect. Dis..

[25] Rodríguez-Pastor R., Escudero R., Lambin X., Vidal M.D., Gil H., Jado I., Luque-Larena J.J., Mougeot F. (2019). Zoonotic pathogens in fluctuating common vole (*Microtus arvalis*) populations: Occurrence and dynamics. Parasitology.

[26] Joannon A., Vialatte A., Vasseur C., Baudry J., Thenail C., Rossing W.A.H., Poehling H.M., Van Helden M. (2008). Combining studies on crop mosaic dynamics and pest population dynamics to foster biological control. Landscape Management for Functional Biodiversity.

[27] Veres A., Petit S., Conord C., Lavigne C. (2013). Does landscape composition affect pest abundance and their control by natural enemies? A review. Agric. Ecosyst. Environ..

[28] Benton T.G., Vickery J.A., Wilson J.D. (2003). Farmland biodiversity: Is habitat heterogeneity the key?. Trends Ecol. Evol..

[29] Chase J.M., Blowes S.A., Knight T.M., Gerstner K., May F. (2020). Ecosystem decay exacerbates biodiversity loss with habitat loss. Nature.

[30] Balmori-de la Puente A., Ventura J., Miñarro M., Somoano A., Hey J., Castresana J. (2022). Divergence time estimation using ddRAD data and an isolation-with-migration model applied to water vole populations of *Arvicola*. Sci. Rep..

[31] Delattre P., Giraudoux P., Baudry J., Musard P., Toussaint M., Truchetete D., Stahl P., Poule M.L., Artois M., Damange J.P. (1992). Land use patterns and types of common vole (*Microtus arvalis*) population kinetics. Agric. Ecosyst. Environ..

[32] Giraudoux P., Delattre P., Habert M., Quéré J.P., Deblay S., Defaut R., Duhamel R., Moissenet M.F., Salvi D., Truchetet D. (1997). Population dynamics of fossorial water vole (*Arvicola terrestris scherman*): A land use and landscape perspective. Agric. Ecosyst. Environ..

[33] Viel J.F., Giraudoux P., Abrial V., Bresson-Andi S. (1999). Water vole (*Arvicola terrestris scherman*) density as a risk factor for human alveolar echinococcosis. Am. J. Trop. Med. Hyg..

[34] Espí A., Del Cerro A., Somoano A., García V., Prieto J.M., Barandika J.F., García-Pérez A.L. (2017). Borrelia burgdorferi sensu lato prevalence and diversity in ticks and small mammals in a Lyme borreliosis endemic Nature Reserve in North-Western Spain. Incidence in surrounding human populations. Enferm. Infecc. Y Microbiol. Clin..

[35] Jacob J., Imholt C., Caminero-Saldaña C., Couval G., Giraudoux P., Herrero-Cófreces S., Horváth G., Luque-Larena J.J., Tkadlec E., Wymenga E. (2020). Europe-wide outbreaks of common voles in 2019. J. Pest Sci..

[36] Somoano A., Ventura J., Miñarro M. (2017). Continuous breeding of fossorial water voles in northwestern Spain: Potential impact on apple orchards. Folia Zool..

[37] Halliez G., Renault F., Vannard E., Farny G., Lavorel S., Giraudoux P. (2015). Historical agricultural changes and the expansion of a water vole population in an Alpine valley. Agric. Ecosyst. Environ..

[38] Berthier K., Galan M., Foltete J.C., Charbonnel N., Cosson J.F. (2005). Genetic structure of the cyclic fossorial water vole (*Arvicola terrestris*): Landscape and demographic influences. Mol. Ecol..

[39] Somoano A. (2020). The role of the montane water vole (*Arvicola scherman*) as a crop pest in NW Spain: Since when?. Galemys.

[40] Miramontes-Carballada Á. (2014). Los paisajes agrarios de España: La evolución de la nueva horticultura y cultivos especializados frente a la agricultura tradicional en la España Atlántica. Rev. De Geogr. E Ordenam. Do Territ..

[41] Baudry J., Bunce R.G.H., Burel F. (2000). Hedgerows: An international perspective on their origin, function and management. J. Environ. Manag..

[42] Le Cœur D., Baudry J., Burel F., Thenail C. (2002). Why and how we should study field boundary biodiversity in an agrarian landscape context. Agric. Ecosyst. Environ..

[43] Couval G., Truchetet D. (2014). Le concept de lutte raisonnée: Combiner des méthodes collectives contre le campagnol terrestre afin de conserver une autonomie furragère. Fourrages.

[44] Gauffre B., Berthier K., Inchausti P., Chaval Y., Bretagnolle V., Cosson J.F. (2014). Short-term variations in gene flow related to cyclic density fluctuations in the common vole. Mol. Ecol..

[45] Garrido-Garduño T., Téllez-Valdés O., Manel S., Vázquez-Domínguez E. (2015). Role of habitat heterogeneity and landscape connectivity in shaping gene flow and spatial population structure of a dominant rodent species in a tropical dry forest. J. Zool..

[46] Anderson S.J., Kierepka E.M., Swihart R.K., Latch E.K., Rhodes O.E. (2015). Assessing the permeability of landscape features to animal movement: Using genetic structure to infer functional connectivity. PLoS ONE.

[47] Russo I.R.M., Sole C.L., Barbato M., Von Bramann U., Bruford M.W. (2016). Landscape determinants of fine-scale genetic structure of a small rodent in a heterogeneous landscape (Hluhluwe-iMfolozi Park, South Africa). Sci. Rep..

[48] Fernández-Ceballos A. (2005). Análisis De La Distribución De *Arvicola terrestris* a Media Escala. Master’s Thesis.

[49] BOE (2008). Real Decreto 409/2008, de 28 de marzo. Boletín Of. Del Estado.

[50] (2010). Directive 2010/63/UE. Horizontal Legislation on the Protection of Animal Used for Scientific Purposes. Off. J. Eur. Union L.

[51] Hoban S. (2014). An overview of the utility of population simulation software in molecular ecology. Mol. Ecol..

[52] Valcárcel N., Villa G., Arozarena A., Garcia-Asensio L., Caballero M.E., Porcuna A., Domenech E., Peces J.J. (2008). SIOSE, a successful test bench towards harmonization and integration of land cover/use information as environmental reference data. Int. Arch. Photogramm. Remote Sens. Spat. Inf. Sci..

[53] Adriaensen F., Chardon J.P., De Blust G., Swinnen E., Villalba S., Gulinck H., Matthysen E. (2003). The application of least-cost modelling as a functional landscape model. Landsc. Urban Plan..

[54] Sawyer S.C., Epps C.W., Brashares J.S. (2011). Placing linkages among fragmented habitats: Do least-cost models reflect how animals use landscapes?. J. Appl. Ecol..

[55] Foltête J.C., Giraudoux P. (2012). A graph-based approach to investigating the influence of the landscape on population spread processes. Ecol. Indic..

[56] ESRI (1996). Arcview GIS: The Geographical Information System for Everyone.

[57] Fichet-Calvet E., Pradier B., Quéré J.P., Giraudoux P., Delattre P. (2000). Landscape composition and vole outbreaks: Evidence from an eight year study of *Arvicola terrestris*. Ecography.

[58] Stewart W.A., Dallas J.F., Piertney S.B., Marshall F., Lambin X., Telfer S. (1999). Metapopulation genetic structure in the water vole, *Arvicola terrestris*, in NE Scotland. Biol. J. Linn. Soc..

[59] Berthier K., Galan M., Weber A., Loiseau A., Cosson J.F. (2004). A multiplex panel of dinucleotide microsatellite markers for the water vole, *Arvicola terrestris*. Mol. Ecol. Notes.

[60] Glaubitz J.C. (2004). Convert: A user-friendly program to reformat diploid genotypic data for commonly used population genetic software packages. Mol. Ecol. Notes.

[61] Raymond M., Rousset F. (1995). GENEPOP (version 1.2): Population genetics software for exact tests and ecumenicism. J. Hered..

[62] Van Oosterhout C., Hutchinson W.F., Wills D.P., Shipley P. (2004). MICRO-CHECKER: Software for identifying and correcting genotyping errors in microsatellite data. Mol. Ecol. Notes.

[63] Nei M. (1987). Estimation of average heterozigosity and genetic distance from a small number of individuals. Genetics.

[64] Belkhir K., Borsa P., Chikhi L., Raufaste N., Bonhomme F. (1996). GENETIX 4.05, Logiciel Sous Windows^TM^ Pour La Génétique Des Populations. Laboratoire Génome, Populations, Interactions, CNRS UMR 5000.

[65] Goudet J. (1995). FSTAT (version 1.2): A computer program to calculate F-statistics. J. Hered..

[66] Piry S., Alapetite A., Cornuet J.M., Paetkau D., Baudouin L., Estoup A. (2004). GENECLASS2, A software for genetic assignment and first-generation migrant detection. J. Hered..

[67] Paetkau D., Slade R., Burden M., Estoup A. (2004). Genetic assignment methods for the direct, real-time estimation of migration rate: A simulation-based exploration of accuracy and power. Mol. Ecol..

[68] Pritchard J.K., Stephens M., Donnelly P. (2000). Inference of population structure using multilocus genotype data. Genetics.

[69] Earl D.A. (2012). Structure Harvester: A website and program for visualizing Structure output and implementing the Evanno method. Conserv. Genet. Resour..

[70] Guillot G., Mortier F., Estoup A. (2005). Geneland: A computer package for landscape genetics. Mol. Ecol. Notes.

[71] Meirmans P.G. (2012). The trouble with isolation by distance. Mol. Ecol..

[72] Drummond C.S., Hamilton M.B. (2007). Hierarchical components of genetic variation at a species boundary: Population structure in two sympatric varieties of *Lupinus microcarpus* (Leguminosae). Mol. Ecol..

[73] Excoffier L., Lischer H.E. (2010). Arlequin suite ver 3.5, A new series of programs to perform population genetics analyses under Linux and Windows. Mol. Ecol. Resour..

[74] Mantel N. (1967). The detection of disease clustering and a generalized regression approach. Cancer Res..

[75] Bohonak A.J. (2002). IBD (Isolation By Distance): A program for analyses of isolation by distance. J. Hered..

[76] Rousset F. (1997). Genetic differentiation and estimation of gene flow from F-statistics under isolation by distance. Genetics.

[77] Cushman S., Wasserman T., Landguth E., Shirk A. (2013). Re-evaluating causal modeling with Mantel tests in landscape genetics. Diversity.

[78] Ackiss A.S., Bird C.E., Akita Y., Santos M.D., Tachihara K., Carpenter K.E. (2008). Genetic patterns in peripheral marine populations of the fusilier fish Caesio cuning within the Kuroshio Current. Ecol. Evol..

[79] Peakall R., Smouse P.E. (2006). GENALEX 6, Genetic analysis in Excel. Population genetic software for teaching and research. Mol. Ecol. Notes.

[80] Grant A.H., Liebgold E.B. (2017). Color-biased dispersal inferred by fine-scale genetic spatial autocorrelation in a color polymorphic salamander. J. Hered..

[81] Peakall R., Ruibal M., Lindenmayer D.B. (2003). Spatial autocorrelation analysis offers new insights into gene flow in the Australian bush rat, *Rattus fuscipes*. Evolution.

[82] Guivier E., Galan M., Chaval Y., Xuéreb A., Ribas Salvador A., Poulle M.L., Voutilainen L., Henttonen H., Charbonnel N., Cosson J.F. (2011). Landscape genetics highlights the role of bank vole metapopulation dynamics in the epidemiology of Puumala hantavirus. Mol. Ecol..

[83] Melis C., Borg Å.A., Jensen H., Bjørkvoll E., Ringsby T.H., Sæther B.E. (2013). Genetic variability and structure of the water vole *Arvicola amphibius* across four metapopulations in northern Norway. Ecol. Evol..

[84] Palsbøll P.J., Berube M., Allendorf F.W. (2007). Identification of management units using population genetic data. Trends Ecol. Evol..

[85] Piertney S.B., Black A., Watt L., Christie D., Poncet S., Collins M.A. (2016). Resolving patterns of population genetic and phylogeographic structure to inform control and eradication initiatives for brown rats *Rattus norvegicus* on South Georgia. J. Appl. Ecol..

[86] Hanski I., Ovaskainen O. (2003). Metapopulation theory for fragmented landscapes. Theor. Popul. Biol..

[87] Vignieri S.N. (2005). Streams over mountains: Influence of riparian connectivity on gene flow in the Pacific jumping mouse (*Zapus trinotatus*). Mol. Ecol..

[88] Telfer S., Piertney S.B., Dallas J.F., Stewart W.A., Marshall F., Gow J.L., Lambin X. (2003). Parentage assignment detects frequent and large-scale dispersal in water voles. Mol. Ecol..

[89] Le Galliard J.F., Remy A., Ims R.A., Lambin X. (2012). Patterns and processes of dispersal behaviour in arvicoline rodents. Mol. Ecol..

[90] Aars J., Dallas J.F., Piertney S.B., Marshall F., Gow J.L., Telfer S., Lambin X. (2006). Widespread gene flow and high genetic variability in populations of water voles *Arvicola terrestris* in patchy habitats. Mol. Ecol..

[91] Somoano A., Miñarro M., Ventura J. (2016). Reproductive potential of a vole pest (*Arvicola scherman*) in Spanish apple orchards. Span. J. Agric. Res..

[92] Peterman W.E. (2018). ResistanceGA: An R package for the optimization of resistance surfaces using genetic algorithms. Methods Ecol. Evol..

[93] Driscoll D.A., Whitehead C.A., Lazzari J. (2012). Spatial dynamics of the knob-tailed gecko *Nephrurus stellatus* in a fragmented agricultural landscape. Landsc. Ecol..

[94] Saucy F., Schneiter B. (1998). Juvenile dispersal in the vole, *Arvicola terrestris*, during rainy nights: A preliminary report. Bull. De La Société Vaud. Des Sci. Nat..

[95] Giraudoux P., Delattre P., Quéré J.P., Damange J.P. (1994). Structure and kinetics of rodent populations, in a region under agricultural land abandonment. Acta Oecologica.

[96] Hahne J., Jenkins T., Halle S., Heckel G. (2011). Establishment success and resulting fitness consequences for vole dispersers. Oikos.

[97] SADEI (2020). Asturian Society for Economic and Industrial Studies.

[98] Morilhat C., Bernard N., Bournais C., Meyer C., Lamboley C., Giraudoux P. (2007). Responses of *Arvicola terrestris scherman* populations to agricultural practices, and to *Talpa europaea* abundance in eastern France. Agric. Ecosyst. Environ..

[99] Somoano A., Bastos-Silveira C., Ventura J., Miñarro M., Heckel G. (2022). Bocage landscape restricts the gene flow of pest vole populations. Dryad Dataset.

