# Supplementary material for: A Bocage Landscape Restricts the Gene Flow of Pest Vole Populations
Source: Life (Basel). 2022 May 27;12(6):800. doi: 10.3390/life12060800 (PMC9225191; doi:10.3390/life12060800)
Supplement: Supplementary file 1 [file life-12-00800-s001.zip › life-1735302-supplementary.pdf]

Supplementary material

Table S1. Descriptive statistics of 12 microsatellite loci tested in ten fossorial water vole demes in Asturias when data from all specimens were pooled. Number of alleles (N_A), size range of microsatellite alleles, observed heterozygosity (H_O) and expected heterozygosity (H_E) are shown. Significant deviation from Hardy–Weinberg equilibrium ($p < 0.001$) is indicated by an asterisk (*).

Locus	N	N_A	Range	H_O	H_E
AV3	137	8	124–164	0.529 *	0.578
AV8	137	13	296–354	0.616 *	0.860
AV11	137	9	362–394	0.725 *	0.763
AV12	137	9	172–204	0.609 *	0.759
AV13P	137	10	120–164	0.796 *	0.854
AV14	137	9	198–236	0.804 *	0.852
AV15	137	10	184–216	0.642 *	0.815
AT2	137	18	175–227	0.667 *	0.871
AT9	137	4	285–296	0.326 *	0.497
AT13	137	3	143–148	0.369 *	0.531
AT22	137	8	198–232	0.558	0.693
AT24	137	10	127–156	0.580 *	0.709
All loci	137	9.3		0.602	0.732

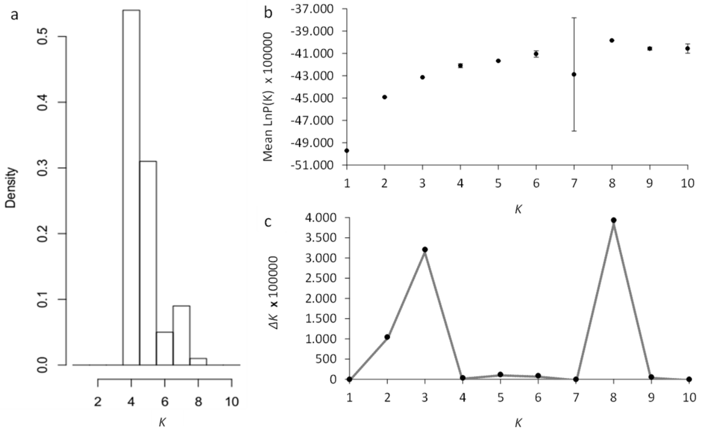

Figure S1. Results from GENELAND analysis: (a) number of populations (*K*) simulated from the posterior distribution. The run giving the highest average posterior probability is shown. Results from STRUCTURE analysis of ten fossorial water vole demes in Asturias: (b) mean ± SD estimated logarithms of probability of data [Ln P (*K*)] against the number of populations tested (*K*); (c) modal value of the second order rate of change of the likelihood function (Δ*K*).

Table S2. Pairwise F_{ST} values (upper semimatrix) and pairwise Euclidean/resistance distances (lower semimatrix, in km) (a), and pairwise F_{ST} values (upper semimatrix) and pairwise landscape suitability/ROMPA (SH/TL) values (lower semimatrix) (b) for the fossorial water vole demes studied in Asturias. Significant F_{ST} values ($p < 0.05$, after standard Bonferroni correction) are indicated in bold.

a

	1	2	3	4	5	6	7	8	9	10
1- Vegadali	0	**0.118**	**0.106**	**0.147**	**0.163**	**0.217**	**0.281**	**0.191**	**0.223**	**0.264**
2- Ceceda	4.21 / 29.78	0	**0.069**	**0.107**	**0.177**	**0.252**	**0.276**	**0.198**	**0.224**	**0.263**
3- Fresnadiello	4.94 / 55.19	1.87 / 20.83	0	**0.105**	**0.143**	**0.188**	**0.206**	**0.147**	**0.173**	**0.196**
4- Poreño	8.78 / 51.65	8.82 / 56.04	7.20 / 70.16	0	**0.102**	**0.196**	**0.217**	**0.150**	**0.180**	**0.232**
5- Serida	12.81 / 51.63	12.81 / 66.58	11.27 / 82.03	4.09 / 25.06	0	**0.188**	0.184	**0.134**	**0.162**	**0.213**
6- Priesca	17.04 / 97.86	15.50 / 100.17	13.74 / 130.59	8.66 / 60.51	6.70 / 53.48	0	0.130	**0.120**	**0.109**	**0.143**
7- Rozada	17.95 / 147.12	17.57 / 150.29	15.76 / 161.71	9.12 / 112.01	5.25 / 100.30	5.19 / 153.30	0	0.092	0.052	0.062
8- Oles	18.69 / 153.04	18.92 / 159.26	17.47 / 161.71	10.15 / 115.81	6.14 / 105.32	8.89 / 132.14	3.83 / 65.05	0	0.071	**0.122**
9- Teleña	19.58 / 151.53	19.54 / 151.47	17.88 / 172.63	10.89 / 113.97	6.80 / 105.34	7.62 / 156.17	2.59 / 39.65	2.18 / 27.50	0	0.003
10- Marina	20.48 / 153.07	20.29 / 156.99	18.63 / 167.56	11.70 / 119.09	7.64 / 107.46	7.57 / 159.73	2.95 / 39.85	3.39 / 32.00	1.08 / 6.67	0

b

	1	2	3	4	5	6	7	8	9	10
1- Vegadali	0	**0.118**	**0.106**	**0.147**	**0.163**	**0.217**	**0.281**	**0.191**	**0.223**	**0.264**
2- Ceceda	10.73 / 45.18	0	**0.069**	**0.107**	**0.177**	**0.252**	**0.276**	**0.198**	**0.224**	**0.263**
3- Fresnadiello	7.64 / 37.75	28.56 / 53.41	0	**0.105**	**0.143**	**0.188**	**0.206**	**0.147**	**0.173**	**0.196**
4- Poreño	4.35 / 38.22	3.81 / 33.59	4.16 / 29.97	0	**0.102**	**0.196**	**0.217**	**0.150**	**0.180**	**0.232**
5- Serida	3.05 / 39.11	3.09 / 39.53	3.43 / 38.64	11.98 / 48.98	0	**0.188**	0.184	**0.134**	**0.162**	**0.213**
6- Priesca	2.10 / 35.80	2.36 / 36.59	2.63 / 36.19	3.83 / 33.16	5.39 / 36.14	0	0.130	**0.120**	**0.109**	**0.143**
7- Rozada	2.07 / 37.16	2.07 / 36.37	2.17 / 34.20	3.42 / 31.15	5.79 / 30.41	6.84 / 35.48	0	0.092	0.052	0.062
8- Oles	2.02 / 37.80	1.94 / 6.75	1.99 / 34.68	3.18 / 32.24	2.91 / 17.89	3.30 / 29.36	3.26 / 12.50	0	0.071	**0.122**
9- Teleña	1.93 / 37.80	1.87 / 36.52	1.90 / 33.94	2.84 / 30.88	3.20 / 21.75	3.62 / 27.56	3.35 / 8.67	4.50 / 11.39	0	0.003
10- Marina	1.82 / 37.28	1.77 / 35.89	1.83 / 34.16	2.45 / 28.67	3.00 / 22.91	3.53 / 26.73	2.73 / 8.04	2.62 / 10.20	13.66 / 20.49	0
